# Therapeutic Potential for Intractable Asthma by Targeting L-Type Amino Acid Transporter 1

**DOI:** 10.3390/biom12040553

**Published:** 2022-04-08

**Authors:** Keitaro Hayashi, Osamu Kaminuma

**Affiliations:** 1 Department of Pharmacology and Toxicology, Dokkyo Medical University School of Medicine, Tochigi 321-0293, Japan; khayashi@dokkyomed.ac.jp; 2 Department of Disease Model, Research Institute of Radiation Biology and Medicine, Hiroshima University, Hiroshima 734-8553, Japan

**Keywords:** L-type amino acid transporter (LAT) 1, steroid-resistant asthma, Th17

## Abstract

Bronchial asthma is a chronic disease characterized by airway inflammation, obstruction, and hyperresponsiveness. CD4^+^ T cells, particularly T helper (Th) 2 cells, and their specific cytokines are important mediators in asthma pathogenesis. However, it has been established that Th subsets, other than Th2, as well as various cell types, including innate lymphoid cells (ILCs), significantly contribute to the development of allergic inflammation. These cells require facilitated amino acid uptake to ensure their full function upon activation. Emerging studies have suggested the potential of pharmacological inhibition of amino acid transporters to inhibit T cell activation and the application of this strategy for treating immunological and inflammatory disorders. In the present review, we explore the possibility of targeting L-type amino acid transporter (LAT) as a novel therapeutic approach for bronchial asthma, including its steroid-resistant endotypes.

## 1. Introduction

It is estimated that more than 300 million people suffer from bronchial asthma worldwide, and the number of patients continues growing [1]. Among the various implicated immunological and inflammatory cells, T helper (Th) 2 cells have been recognized as major players in asthma pathogenesis. The identification of CD4^+^ T cells expressing type 2 cytokines, such as interleukin (IL)-4, IL-5, and IL-13, in bronchial biopsy specimens and bronchial lavage fluids [2,3], as well as the recent clinical application of biomedicines against type 2 cytokines [4,5,6], further emphasizes the importance of Th2 cells in the development of asthma.

Glucocorticoids (steroids) are principally employed in all steps of asthma treatment. Consistent with the steroid-mediated suppression of Th2 cell cytokine production, bronchial asthma is now considered a controllable disease, given the efficacy and safety of inhaled corticosteroid (ICS)-based therapies. However, approximately 10% of patients with asthma fail to adequately respond to steroids and other existing therapeutics [7].

Targeting activated cells refractory to steroid treatment is a promising strategy for resolving issues that continue to hinder asthma therapy. Recently, we discovered the enhanced expression of L-type amino acid transporter (LAT) 1 in activated T cells [8,9]. LAT1 regulates the incorporation of various essential amino acids into cells, potentially contributing to the development of T cell-mediated allergic inflammation. Herein, we describe the possible management of bronchial asthma, including steroid-resistant endotypes by targeting LAT1, summarizing the recent establishment of asthma pathogenesis, including the contribution of LAT1, and efforts to generate a novel LAT1 inhibitor.

## 2. Classification of Asthma Endotypes

### 2.1. Type 2-High (T2) Asthma

Bronchial asthma can be classified into two major categories based on the key biological pathway: T2 and type 2-low (non-T2) [10,11,12]. Naïve CD4^+^ T cells differentiate into Th cell subpopulations such as Th1, Th2, Th9, and Th17 cells, exhibiting characteristic cytokine-producing capacities according to their surrounding environment. Among them, Th2 cells and type 2 cytokines are predominantly involved in mechanisms underlying T2 asthma. Type 2 cytokines, including IL-4, IL-5, and IL-13, drive IgE class switch, eosinophil recruitment into inflammatory sites, and mucus production [13]. In murine models of asthma, diminished airway inflammation was observed in several lines of eosinophil-deficient mice [14,15,16], indicating a considerable contribution of eosinophils to T2 asthma [17,18].

Consistent with the clinical effectiveness of an anti-IgE antibody [19,20], IgE-mediated mast cell activation is a crucial cascade that induces allergic reactions. However, in an allergen-induced airway inflammation model, substantial airway hyperresponsiveness, accompanied by eosinophil infiltration, was induced in mast cell-deficient mice [21]. CD4^+^ T cell depletion following administration of an anti-CD4 antibody suppressed eosinophilic inflammation. Furthermore, the adoptive transfer of in vitro-differentiated Th2 cells to normal mice could induce airway eosinophilic inflammation and hyperresponsiveness following allergen provocation [22]. Given that this procedure is free from allergen-specific IgE synthesis, it is strongly suggested that Th2 cells directly contribute to asthma pathogenesis, independent of the IgE/mast cell cascade. 

Stimulation-induced type 2 cytokine production by Th2 cells is strongly suppressed by steroid application in vitro [23]. Accordingly, most patients with T2 asthma are likely to be steroid-sensitive; thus, decreased serum type 2 cytokine levels can be observed in parallel with symptomatic remission following steroid treatment [24].

In addition to Th2 cells, another cellular source of type 2 cytokines has been identified. Allergens, pollutants, and microorganisms stimulate bronchial epithelial cells to release IL-25, IL-33, and thymic stromal lymphopoietin (TSLP) [25,26,27], which stimulate group 2 innate lymphoid cells (ILC2), and, in turn, release type 2 cytokines [28,29]. In contrast to Th2 cells, steroids do not potently suppress ILC2-mediated type 2 cytokine production [30]. These findings are consistent with the substantial efficacy of biomedicines against type 2 cytokines in patients with steroid-resistant asthma [31].

### 2.2. Non-T2 Asthma

It has been highlighted that Th cell subpopulations that preferentially produce non-type 2 cytokines can also contribute to the development of allergic inflammation without eosinophil accumulation. Th17 cells have been implicated in non-T2 asthma pathology via the production of IL-17A, IL-17E, and IL-22 [32]. IL-17A was shown to be upregulated in airway biopsy specimens and sputa from patients with moderate-to-severe asthma [33,34,35]. Peripheral blood mononuclear cells from patients with severe asthma reportedly exhibit an enhanced capacity to synthesize IL-17A and IL-22 following in vitro activation [36]. In addition, IL-17A stimulated human bronchial fibroblasts to produce pro-fibrotic cytokines [35] and directly increased airway smooth muscle contractility in humans and mice [37]. The adoptive transfer of Th17 cells into normal mice could induce airway hyperresponsiveness, along with neutrophil infiltration following allergen challenge [38]. Notably, steroid treatment failed to effectively suppress Th17-mediated responses [38].

Therefore, as a new strategy to treat steroid-resistant asthma, the potential use of antibodies against IL-17A and its receptor has been investigated. Administration of an anti-IL-17A antibody attenuated house dust mite-induced airway inflammation and hyperresponsiveness in mice [39]. Human anti-IL-17A antibodies (secukinumab and ixekizumab) are currently approved to treat inflammatory diseases other than asthma, such as plaque psoriasis. Notably, brodalumab, a monoclonal antibody against human IL-17 receptor approved for moderate-to-severe plaque psoriasis, failed to demonstrate the potent efficacy in patients with moderate-to-severe asthma in the first clinical trial [40]; however, further investigations with adequate recruitment of Th17 cell-dependent endotype patients should be considered.

In addition, the involvement of immunological and inflammatory cells other than Th17 cells has been suggested in the development of non-T2 and steroid-resistant asthma. Along with Th2 and Th17 cells, an increased number of other T cells, such as Th1 and Th9 cells, was detected in the lungs of patients with asthma [41,42]. Both Th1 and Th9 cells could potentially induce significant airway hyperresponsiveness in normal mice following adoptive transfer and allergen challenge [22,23,43,44,45]. Regardless of differences in the dominant cellular populations accumulated in the lungs, e.g., neutrophils induced by Th1 and Th17 and eosinophils and neutrophils induced by Th9 cells, most non-T2 responses tend to exhibit steroid resistance [23,38].

## 3. LAT1 and Its Inhibitors

Amino acid delivery into cells is regulated via transporters, termed solute carriers (SLCs). Among >60 amino acid transporters with differential specificity and potency in humans [46], LAT1 (SLC7A5) transports large neutral amino acids, mostly classified as essential amino acids, including branched-chain amino acids (Leu, Ile, Val), aromatic amino acids (Phe, Tyr, Trp, His), and methionine in a sodium-independent manner [47]. LAT1 forms a complex with CD98 (SLC3A2/4F2 heavy chain) to achieve its stable localization at the plasma membrane [48]. The biological significance of LAT1 has been reported, particularly its gene targeting strategy. Reportedly, *Slc7a5*-null mouse embryos display neural and limb bud outgrowth defects [49]. Additionally, *Slc7a5*-deficient mice displayed enhanced osteoclastogenesis and bone loss [50]. Notably, LAT1 is preferentially expressed in diverse human cancer cells [51,52]. Accumulating evidence suggests that LAT1 inhibition can effectively regulate the growth of various tumor cells [51]. Knockdown of human LAT1 using small interfering RNAs markedly downregulated the proliferation of MIA Paca-2 (pancreatic cancer), LNCaP and C4-2 (prostate cancer), HEC1A (endometrial cancer), and MKN-45 (gastric cancer) cells in vitro [53,54,55,56]. In addition, substantial in vivo tumor growth inhibition was observed following inoculation with anti-human LAT1 antibody-generating hybridoma cells [57]. Furthermore, oncogenic *KRAS* gene mutant-mediated colorectal tumor formation was alleviated in *Slc7a5*-knockout mice [58]. Given these findings, LAT1 has attracted worldwide attention as a novel target for cancer treatment. 

**Figure 1:**
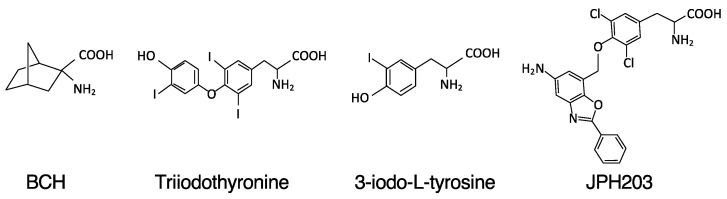
Structures of LAT1 inhibitors. BCH (2-aminobicyclo-(2,2,1)-heptane-2-carboxylic acid) inhibits all L-type amino acid transporters. Triiodothyronine is a thyroid hormone that exerts an inhibitory effect on LAT1. Additionally, 3-iodo-L-tyrosine was identified as a LAT1 inhibitor by comparative modeling and computational screening. JPH203 is a high-affinity LAT1-specific inhibitor.

For the clinical application of a LAT1-targeting strategy, researchers have attempted to produce LAT1 inhibitors (Figure 1). In the late 1960s, 2-aminobicyclo-(2,2,1)-heptane-2-carboxylic acid (BCH) was identified as an amino acid transporter inhibitor. BCH was shown to inhibit LAT1; however, a markedly high concentration is required to mediate this action. Furthermore, BCH lacked selectivity toward LAT1; thus, all isoforms of system L transporters (LAT1, LAT2 LAT3 and LAT4) could be suppressed. Geier et al. identified 3-iodo-L-tyrosine, 3,5-diiodo-L-tyrosine, fenclonine, and acivicin as potential LAT1 inhibitors using comparative modeling and computational screening [59]. Triiodothyronine, a thyroid hormone, exerts an inhibitory effect on LAT1 with a relatively high affinity and selectivity [60]. Based on the T3 structure, Endou et al. generated a series of compounds exhibiting high LAT1 affinity. Among them, JPH203 (KYT-0353: (S)-2-amino-3-(4-((5-amino-2-phenylbenzo[d]oxazol-7-yl) methoxy)-3,5-dichlorophenyl) propanoic acid) showed potent and selective inhibitory effects against LAT1 [61] (Figure 1). In HT-29 cells, the half-maximal inhibitory concentrations (IC_50s_) of JPH203 were 0.06 and 4.1 µM for inhibiting leucine uptake and cell growth, respectively; however, at nearly the same dose range, JPH203 did not impact LAT2, indicating the substantial selectivity of JPH203 for LAT1. The significant efficacy of JPH203 has been demonstrated in cancer cell transplantation experiments in vivo [61]. Based on these findings, a first-in-human study for treating advanced solid tumors was completed, which confirmed the efficacy and safety of this compound [62].

## 4. LAT1 in T Cells

**Figure 2:**
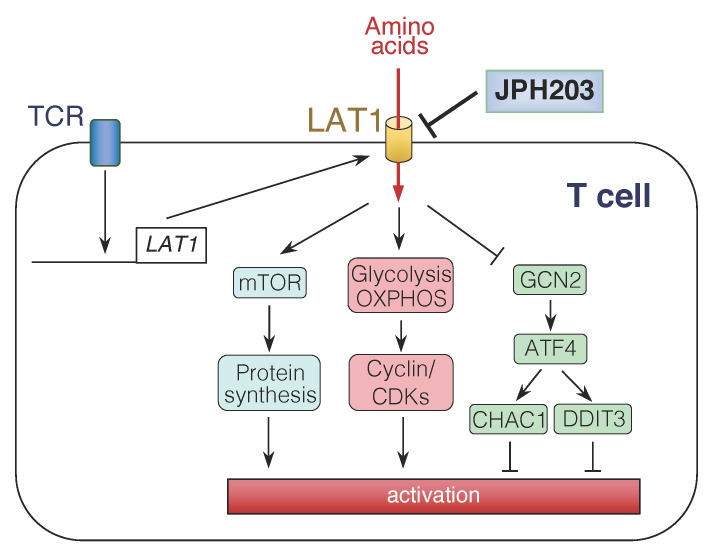
Regulation of T cell activation by L-type amino acid transporter (LAT) 1. In activated T cells, LAT1 is upregulated to facilitate amino acid uptake. JPH203, a LAT1-specific inhibitor, attenuates mechanistic target of rapamycin (mTOR) and metabolic reaction, and contrary, activates activating transcription factor (ATF) 4, leading to inhibition of T cell activation. CHAC1: ChaC glutathione specific gamma-glutamylcyclotransferase 1, CDK: cyclin-dependent kinase, DDIT3: DNA damage-inducible transcript 3, GCN2: general control nonderepressible 2, OXPHOS: oxidative phosphorylation, TCR: T cell receptor.

Activation of T cells following the recognition of allergen epitopes through T cell receptors (TCRs), accompanied by the participation of co-stimulatory molecules, facilitates nutrient uptake from the extracellular environment [63]. Targeted disruption of the *Slc7a5* gene in CD4^+^ T cells did not affect T cell selection and homeostasis of the naïve T cell pool in the thymus [64], which is consistent with negligible LAT1 expression in naïve T cells [8]. However, LAT1 plays a substantial role in activated T cells (Figure 2). Stimulation of TCRs induced marked LAT1 expression in human T cells [8]. Given the suppression of leucine uptake, JPH203 could inhibit stimulation-induced production of IL-4, IL-5, IL-17, and interferon (IFN)-γ, as well as the proliferation of human and/or mouse T cells [8,9]. The blockade of amino acid incorporation into T cells downregulated the mechanistic target of rapamycin (mTOR)-dependent signaling pathway, oxidative phosphorylation (OXPHOS), glycolysis, and expression of cyclin and cyclin-dependent kinases [9]. Furthermore, upregulation of ChaC glutathione-specific gamma-glutamylcyclotransferase 1 and DNA damage-inducible transcript 3 by activating transcription factor 4, an amino acid starvation-inducible transcription factor, contributed to the suppression of T cell activation following JPH203 treatment [8,65] (Figure 2).

## 5. LAT1 in Allergic and Inflammatory Diseases

We hypothesized that the LAT1 inhibitor has a beneficial effect for treating allergic diseases in which activated T cells play a crucial role. We have developed murine models of allergic inflammation in various target tissues and demonstrated the essential contribution of allergen-specific T cells to their pathogenesis [66]. Consistent with the enhanced expression of LAT1 in skin-accumulated CD4^+^ T cells in patients with atopic dermatitis, JPH203 treatment could suppress allergen-induced skin inflammation in both ovalbumin (OVA)-immunized and OVA-specific Th2 cell-transferred mice [9]. Upregulation of LAT1 in CD4^+^ T cells was observed in imiquimod-induced psoriasis, another skin inflammation model [67]. JPH203 treatment and *Slc7a5* gene disruption in CD4^+^ T cells alleviated disease progression with cytokine production in a psoriasis mouse model [67]. In addition, JPH203 could effectively suppress allergen-induced airway and nasal hyperresponsiveness in immunized and/or Th2-transferred mice [65,68]. Interestingly, JPH203 treatment ameliorated eosinophil accumulation in the nasal mucosa but not in the lungs [65,68]. Pharmacological inhibition of LAT1 is a novel promising strategy to control allergic and inflammatory disorders. 

## 6. Possible Management of Steroid-Resistant Asthma by LAT1 Inhibitors

**Figure 3:**
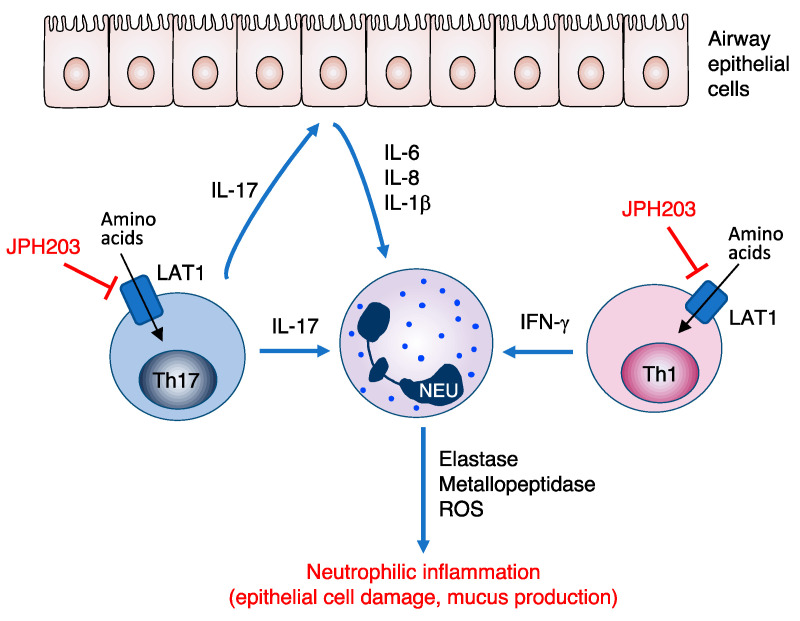
Possible management of steroid-resistant asthma by JPH203. T helper (Th) 17 and Th1 cells activate neutrophils, which causes progression of steroid-resistant asthma. JPH203 potentially terminates the disease cascade by starving amino acids in those cells. LAT1: L-type amino acid transporter 1, ROS: reactive oxygen species, NEU: neutrophil.

In addition to improving Th2-driven asthma phenotypes in mice, we observed that JPH203 could effectively suppress IL-17A production by human T cells [8]. Therefore, pharmacological LAT1 inhibition may afford therapeutic benefits against steroid-resistant asthma, at least when mediated via Th17 cells. We are currently investigating the effect of JPH203 on allergic airway inflammation in mice transferred with in vitro-differentiated Th17 cells. Preliminary findings indicated that JPH203 administration suppressed allergen-induced airway hyperresponsiveness, neutrophil accumulation, and IL-17 production in the lungs, whereas dexamethasone treatment failed to demonstrate any significant improvement (manuscript in preparation). On the other hand, targeted deletion of LAT1 in skin-resident RORγt-expressing cells containing Th17 cells and ILCs significantly improved imiquimod-induced psoriasis in mice [67]. JPH203 also suppressed IFN-γ production by activated human T cells [8], suggesting the involvement of LAT1 in other steroid-resistant mechanisms (Figure 3).

## 7. Conclusions

Despite efforts to develop effective treatments, a suitable strategy to control steroid-resistant asthma has not been successfully established. The technique of depleting the nutrients in T cells, distinct from current treatment strategies, could potentially modulate steroid-resistant mechanisms. Phase II clinical trials assessing JPH203 for cancer treatment are ongoing, highlighting its safety and usefulness. Further in-depth investigations evaluating JPH203, including its efficacy, pharmacokinetics, and formulations, are warranted to establish its potential application for managing intractable asthma.
